# Sensitivity and Bias in Decision-Making under Risk: Evaluating the Perception of Reward, Its Probability and Value

**DOI:** 10.1371/journal.pone.0033460

**Published:** 2012-04-06

**Authors:** Madeleine E. Sharp, Jayalakshmi Viswanathan, Linda J. Lanyon, Jason J. S. Barton

**Affiliations:** 1 Human Vision and Eye Movement Laboratory, Departments of Medicine (Neurology), University of British Columbia, Vancouver, British Columbia, Canada; 2 Ophthalmology and Visual Sciences, University of British Columbia, Vancouver, British Columbia, Canada; 3 Psychology, University of British Columbia, Vancouver, British Columbia, Canada; University of Leicester, United Kingdom

## Abstract

**Background:**

There are few clinical tools that assess decision-making under risk. Tests that characterize sensitivity and bias in decisions between prospects varying in magnitude and probability of gain may provide insights in conditions with anomalous reward-related behaviour.

**Objective:**

We designed a simple test of how subjects integrate information about the magnitude and the probability of reward, which can determine discriminative thresholds and choice bias in decisions under risk.

**Design/Methods:**

Twenty subjects were required to choose between two explicitly described prospects, one with higher probability but lower magnitude of reward than the other, with the difference in expected value between the two prospects varying from 3 to 23%.

**Results:**

Subjects showed a mean threshold sensitivity of 43% difference in expected value. Regarding choice bias, there was a ‘risk premium’ of 38%, indicating a tendency to choose higher probability over higher reward. An analysis using *prospect theory* showed that this risk premium is the predicted outcome of hypothesized non-linearities in the subjective perception of reward value and probability.

**Conclusions:**

This simple test provides a robust measure of discriminative value thresholds and biases in decisions under risk. *Prospect theory* can also make predictions about decisions when subjective perception of reward or probability is anomalous, as may occur in populations with dopaminergic or striatal dysfunction, such as Parkinson's disease and schizophrenia.

## Introduction

How humans make decisions is an important question in the study of human behavior and cognition. The manner in which options are weighed and different forms of information incorporated remain poorly understood, as are the systematic biases or misperceptions that lead to decisions that deviate from rational behavior. However, progress has been made in the study of decisions involving risks [1] , and the neural circuitry underlying the influences of reward on behavior are being elucidated, particularly in regard to the role of dopaminergic systems and the basal ganglia [2], [3], [4]. Ultimately, our understanding of decision-making in such situations may clarify some important aspects of cognitive dysfunction in conditions with disorders of these systems, such as Parkinson's disease, iatrogenic pathologic gambling, and schizophrenia [5], [6].

While many reports on motivation have examined how reward modulates responses, resulting for example in faster or more accurate saccades [7], [8], [9], [10], [11], it is also important to understand how subjects make decisions when choices involve rewards, and what factors guide those decisions. Decision-making has been defined by a few key parameters: the likelihood of an outcome (probability), the size of the outcome (magnitude) and the variance of the outcomes [12]. The context of the decision-making exercise is also an important parameter, particularly whether the choice is presented under ‘risk’, when the probabilities of gains or loss are explicitly defined, or ‘ambiguity’, when the probabilities of outcomes are not known to the subject [1], [13].

Much research has focused on creating models that examine the different variables involved in decision-making. However, few of the tasks used in these studies have been described in terms of their internal validity with the purpose of developing them for use in the clinical setting. There are currently only a few clinical tools to assess decisions involving rewards or penalties. The most widely used is the Iowa Gambling Task [14], [15]. On this test, subjects begin without knowledge of the probabilities of gains or losses involved and must discover these on their own over the course of the test – hence it assesses decisions under *ambiguity* and subsequent learning. While the Iowa Gambling Task has had success predicting naturalistic risk-taking behaviours, others have pointed out that this test does not lend itself well to decomposition - that is, the isolation of the specific cognitive components involved: i.e. “it is almost impossible to determine the degree to which individual differences in behavior in the Iowa Gambling Task reflect differences in learning, risk attitudes, and/or sensitivity to gain and/or loss magnitude” (p. 14) [16]. Similar criticisms have been levied against the Balloon Analogue Risk Task [17]. As a consequence, there is a need for new experimental paradigms that have external validity for natural behaviour, are emotionally engaging, and can decompose performance into variables related to risk-taking [16].

In this experiment, our main goal was to create a simple economic scenario to model decision-making under risk. It is not clear whether risk and ambiguity lie at two extremes of a continuum or whether they involve distinct neural processes [13], [18], [19]. Given the latter possibility, a clinical test of decision-making under risk may be a useful complement to evaluations of decision-making under ambiguity. In addition, one advantage of tests of decision-making under risk is that the use of explicitly defined probabilities allows for mathematical decomposition of the decision-making process into its different cognitive constructs.

Our paradigm required subjects to choose between two prospects differing in the size and probability of reward, to maximize their financial gain. On each trial we varied the difference in value of the two prospects to create a spectrum of choices. We then analyzed the decisions of subjects in this two-alternative forced-choice paradigm by using traditional concepts from the field of psychophysics to determine two key summary variables: the discriminative threshold, which reflects the difference in value between the two prospects that is required to cause subjects to choose one prospect reliably more than the other, and the choice bias, which is the difference in value at which a subject is equally likely to choose either prospect. Choice bias may be an important clinical parameter, as it can show whether behaviour is risk-averse or risk-seeking, by revealing whether subjects are more likely to choose the high-probability but low-yield prospect or the low-probability but high-yield one. Thus we anticipate that such summary variables may prove to be useful characterizations of group performance in future studies of clinical populations.

Our initial analyses used the framework of *expected value theory*, which posits that subjects decide rationally by computing the objective worth of the prospects, expected value being the product of reward magnitude times reward probability. However, although many studies use expected value to characterize prospects [7], [20], [21], human decisions are not always marked by rationality. A second goal of our work was to evaluate our results using *prospect theory*, which holds that decisions are made on the basis of perceived value rather than objective worth, and that perceived magnitude and perceived probability of reward have non-linear relationships with their objective counterparts [22]. Our results show that some of the irrational anomalies we discovered using expected value terms can be explained by the non-linear functions developed by others using *prospect theory*.

## Materials and Methods

### Subjects

Nineteen subjects (10 female, 9 male; 28–45 years of age, all right-handed) participated, all healthy with no prior psychiatric or neurological illness, not on medication other than the oral contraceptive, and with normal or corrected-to-normal vision. Subjects were surveyed for their caffeine intake (mean 1.0 cups, s.d. 1.1) and the number of hours of sleep obtained the previous night (mean 6.9 h, s.d. 1.2). Subjects were also screened for pathological gambling using the South Oaks Gambling Screen [23] and none were found to be gamblers (mean score 0.53, s.d. 1.01).

### Ethics

The institutional review boards of Vancouver General Hospital and the University of British Columbia approved the protocol, and all subjects gave written informed consent in accordance with the declaration of Helsinki. Subjects were paid $10.00 for participation and received additional payment for rewards gained during the experiment ($0.20 per coin won), with payments ranging from $36.40 to 56.20.

### Apparatus and protocol

Subjects sat in dim illumination 57 cm away from 22″ CRT screen, with their head position maintained by a chin-rest and viewing with both eyes. Screen resolution was 1024 by 768 pixels, which covered 39° and 30° of visual field, respectively. Eye movements were recorded by a video-based system using the pupil and the corneal infrared-light reflex to estimate gaze position (Eyelink 1000 from SR Research Ltd, Mississauga, Canada). Stimuli, trials and experimental blocks were created using SR Research Experiment Builder 1.1.2.

**Figure 1:**
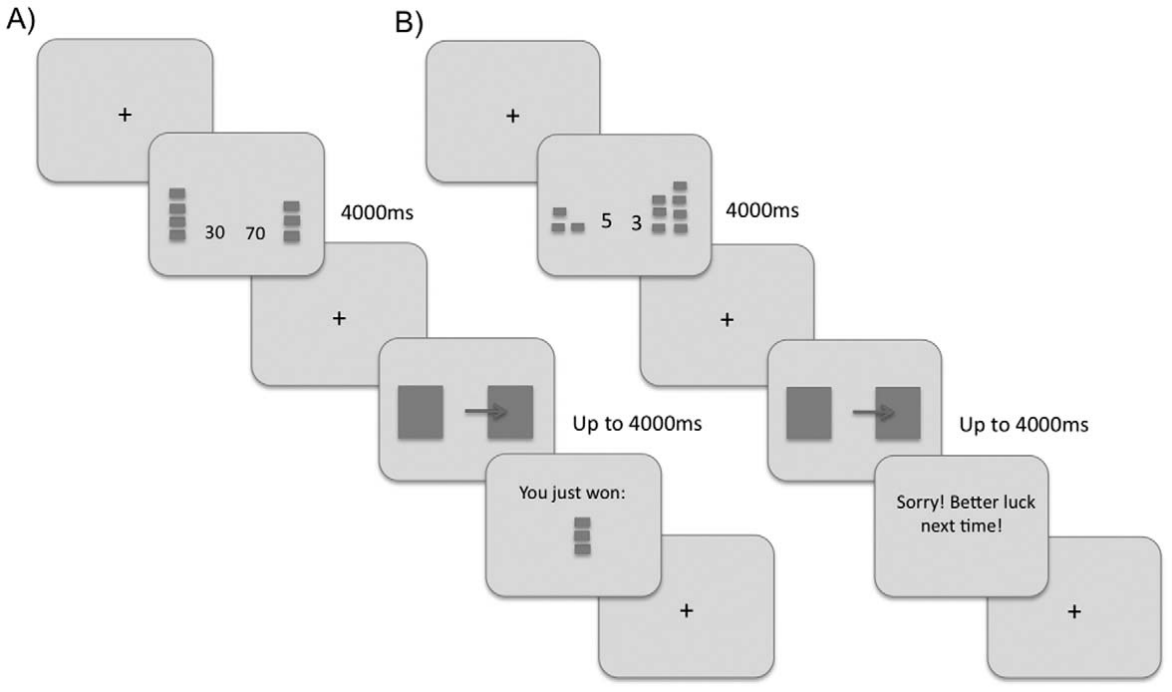
Two examples of trial sequences. Each panel shows a screen display, with the series of screens in a trial progressing from top left to bottom right. A). First version. A cross at screen center appears, which the subject must fixate first. The next screen is an information screen showing the outcomes of the left and the right prospects. Here probability is represented as a number (in this example, 30% chance of reward on the left, 70% chance on the right) and magnitude of reward is shown pictorially (4 tokens on the left, 3 tokens on the right, each token worth $0.20). After 4 sec, this is replaced by another central cross, once subjects fixate this, a choice screen appears for up to 4 sec, and subjects make a saccade into one of the boxes to indicate their choice. The computer then determines with the probability of the prospect chosen whether the subject gets a reward. In this example the subject received 3 tokens. This is then replaced by the fixation cross for the next trial. B). Second version. This is similar except that probability is represented pictorially (3 rectangles for 30% versus 7 for 70%) and magnitude numerically (5 tokens on the left, 3 tokens on the right). In this example, the subject did not get a reward.

Our strategy was to have subjects choose between two prospects, one on the left and one on the right side of the screen, each of which had a certain magnitude and probability of reward, differing from each other and differing from trial to trial. At the beginning of the experiment, subjects were instructed that the task was similar to a game show in which they were to maximize their gain by choosing between two ‘mystery boxes’. Each trial (Figure 1) began with a 1-second view of a white screen with a dark fixation cross at screen center. This was followed by an information screen that showed the reward magnitude and probability for the prospect on the right and that for the prospect on the left. This remained visible for 4 seconds, during which time subjects were free to move their gaze as they deliberated on their choice. After 4 seconds the information screen was replaced by another central fixation cross, and after subjects had achieved fixation within 4° of the center, this screen was followed by a display showing two boxes, which were squares of 8° width, one centered 6° left of fixation and the other centered 6° right of fixation, each with a fixation cross at the center of the square. Subjects were instructed to make a saccade to the centre of the box corresponding to their choice. (Our experimental paradigm yielded similar data when a manual response was used instead in an older cohort – see Appendix S2). If no saccade was performed into one of the choice boxes within 4 seconds, then the trial was terminated and recycled to reappear later during the experiment. If a response into a choice box was made, the computer used the probability and magnitude information of the choice made by the subject to determine if they received a reward or not. This information was conveyed to the subject by a feedback screen with a message stating “Sorry! Better luck next time” if no reward was given or “You just won x coins” if they won the gain at stake. After 1.5 seconds this disappeared and the fixation cross appeared for the next trial. Though subjects were not required to learn any contingencies during the task, we attempted to minimize any learning effect induced by providing trial-by-trail feedback by explicitly telling subjects prior to starting the experiment that the probabilities depicted were, in fact, true probabilities and that their decision on one trial would not affect the outcome on a subsequent trial.

A rational decision-maker would calculate expected value (EV), which is simply the magnitude of reward multiplied by the probability of reward, and choose the prospect with the higher expected value. In humans, though, there is some stochastic variability in choice [24], and how this is reflected in discriminative sensitivity to expected value is one of the goals of this study. Thus the key factor in each trial is the balance between the expected value of one prospect versus that of the other. In our experimental conditions, we required subjects to trade off between one prospect with higher reward probability (which we arbitrarily designated as Prospect 1) and a second prospect with higher reward magnitude (Prospect 2). We expressed the difference in expected value (EV) between the two prospects as (EV1–EV2)/[(EV1+EV2)/2], which we called the *EV-ratio*. By our arbitrary convention a positive EV-ratio indicates that the more favorable prospect is the one with higher reward probability, whereas a negative EV-ratio indicates that a rational subject should choose the prospect with the larger size of reward. We created 14 different combinations, with the sizes of EV-ratio ranged from 10%, a difficult discrimination in which the odds are 1.11∶1, to 90%, an easy discrimination corresponding to odds of 2.7∶1 (Table 1).

**Table 1:** Probabilities and magnitudes of reward for the two prospects in each trial, for the 14 experimental conditions and the 3 control conditions.

Experimental Conditions						
Reward Magnitude (coins)		Reward Probability (%)		EV	EV	EV Ratio
Prospect 1	Prospect 2	Prospect 1	Prospect 2	Prospect 1	Prospect 2	
1	4	60	40	0.6	1.6	−0.909
1	5	70	30	0.7	1.5	−0.727
1	4	70	30	0.7	1.2	−0.526
2	5	60	40	1.2	2.0	−0.500
1	3	70	30	0.7	0.9	−0.250
1	5	80	20	0.8	1.0	−0.222
3	5	60	40	1.8	2.0	−0.105
3	4	60	40	1.8	1.6	0.117
4	5	60	40	2.4	2.0	0.182
1	3	80	20	0.8	0.6	0.286
2	3	70	30	1.4	0.9	0.435
3	4	70	30	2.1	1.2	0.545
1	2	80	20	0.8	0.4	0.667
2	3	80	20	1.6	0.6	0.909

Control conditions						
Reward Magnitude (coins)		Reward Probability (%)		EV	EV	EV Ratio
Prospect 1	Prospect 2	Prospect 1	Prospect 2	Prospect 1	Prospect 2	
2	4	50	50	1.0	2.0	−0.667
3	3	70	30	2.1	0.9	0.800
5	2	60	40	3.0	0.8	1.158

Prospect 1 was arbitrarily designated as having the higher reward probability (EV = expected value = probability X magnitude).

We also added 3 control combinations in which a) one prospect had both greater magnitude and greater probability of reward than the other, b) probability differed between the two but magnitude was equal, or c) magnitude differed but probability was equal (Table). In these control trials it is obvious which prospect is the better choice, as there is no need to trade off probability against magnitude. Control trials verified that subjects understood the task and were attempting to maximize gain.

As each information screen has to convey simultaneously the magnitude and probability of reward at risk, to avoid confusion we depicted one pictorially and the other numerically. Two versions of the experiment were created. In the first, reward magnitude was depicted as a stack of one to five rectangular tokens, with each token worth $0.20, and reward probability by a percentage number (20 to 80). In the second, reward magnitude was represented by a number and reward probability by a stack of two to eight rectangles, to indicate probabilities ranging from 20 to 80%. Ten subjects were assigned to the first version and ten to the second.

The experiment consisted of 5 blocks separated by a rest break. Each block consisted of the same set of 34 trials. Each of the 17 different trials (14 experimental combinations and 3 control combinations) was shown twice in a block, once with the higher expected value on the right and once with it on the left. The order of the trials was randomized within each block. At the end of each block subjects were told how much money they had won. After the end of the experiment, subjects were paid the gains they had accrued.

### Data Analysis

**Figure 2:**
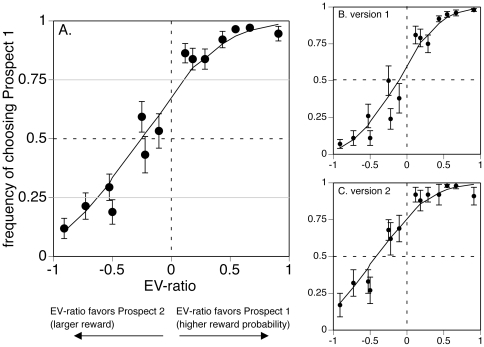
Results. The frequency of choosing Prospect 1 (the prospect with higher reward probability) is plotted as a function of the EV-ratio for (graph A) all subjects, (graph B) subjects in Version 1, in which probability is represented numerically – see Figure 1A, and (graph C) subjects in Version 2 in which probability is represented pictorially – see Figure 1B. EV-ratio>0 indicates that Prospect 1 with the higher reward probability also has the higher EV-ratio; EV-ratio<0 indicates that Prospect 2 with the larger reward is the better choice. The point of equivalence (when frequency of choosing higher probability is equal to frequency of choosing larger reward, i.e. the dotted horizontal line showing frequency = 0.5) should occur when the EV-ratio = 0 (dotted vertical line) in a rational decision-maker, but this is shifted in our subjects to the left, indicating greater tendency to choose Prospect 1, which has the higher reward probability. This is more so in Version 2 than Version 1 (graph A vs. B), indicating that there is an additional bias in favour of the property depicted pictorially. In (A), the solid grey lines indicate thresholds for 25% and 75% frequency of choice: half the distance on the x-axis covered by the fitted curve between these two frequencies is taken as our discriminative threshold. Error bars are one standard error.

The main variable was the choice made by the subject, which we operationalized as the frequency of choosing the prospect with higher probability. This was plotted as a function of the EV-ratio. We first analyzed choice with repeated-measures ANOVA with main factors of EV-ratio and experiment version (pictorial magnitude/numerical probability, numerical magnitude/pictorial probability), and subject as a random effect. Second, we fit curves to the data, using least-squares linear regression of normalized (z-transformed) frequency-of-response data [25]. This was done for individual subject data for statistical purposes, and on average group data for illustrative purposes in Figure 2. From these curves we first obtained the point of equivalence for each subject, the EV-ratio at which a subject is equally likely to choose either prospect (i.e. 50% likelihood of choosing the side with higher probability). We then tested the hypothesis that the point of equivalence was significantly different from an EV-ratio of zero, which would indicate a systematic bias in choice. Second, we obtained estimates for the EV-ratios at which subjects had a 25% and a 75% likelihood of choosing the side with higher probability. Half of the difference between these two EV-ratios is equivalent to a 75% discriminative threshold for choice, or ‘just noticeable difference’ in EV-ratio, which is midway between random guessing (50% likelihood) and certainty (0% or 100% likelihood).

## Results

On the 30 control trials, 18 of the 19 subjects reliably chose the side with higher expected value, with 2 subjects making 2 errors, 3 subjects making 1 error and 13 subjects making no errors. One subject only chose the correct side 60% of the time, which was no better than chance. Her data are excluded from the following analysis, because we cannot be certain that her decisions are guided by a desire to maximize reward.

In the experimental trials, the ANOVA showed a main effect of EV-ratio (F(13,221) = 65.7, p<.0001), confirming a robust and consistent effect of EV-ratio on choice. The curve fit to group data showed a bias towards choosing the side with higher probability rather than higher reward (Figure 2A). The point of equivalence for these group-averaged data occurred at an EV-ratio of −0.25. Analyzing the individual subject data, the mean point of equivalence was −0.38 (s.d. 0.67), which was significantly different from an EV-ratio of 0 (t(18) = 2.40, p<.028), with 95% confidence interval of [−0.71, −0.05]. Thus, the side with greater reward had to have a 38% larger expected value than the side with higher probability for subjects to be indifferent in their decision. This indicates a statistically significant degree of risk aversion in this group of subjects.

There was an interaction between session and EV-ratio (F(13,221) = 2.05, p<.02). Subjects were more likely to choose the side with larger reward when reward magnitude was depicted pictorially and reward probability numerically (Figures 1A, 2B), with an equivalence point of −0.13, than when the reverse was true, where the equivalence point was −0.65 (Figures 1B, 2C). Thus the pictorial symbol has a 26% advantage over Arabic numbers in biasing choice. The slopes of the curves fitted to the two sessions did not differ, indicating that sensitivity to expected value did not differ by which property was indicated pictorially and which numerically.

For the curve fit to group data, the discriminative threshold for EV-ratio, was 0.36. Analyzing the individual subject data, the mean of individual discriminative thresholds was 0.43 (s.d. 0.34), giving a 95% confidence interval of [0.27, 0.59].

The above analysis in the traditional framework of *expected value theory* assumes that subjects have veridical estimates of value and probability. *Prospect theory* holds that neither is true, and that subjective perception of reward magnitude and probability are non-linear functions. Recent work has summarized much normative data focused on finding the functions that best fit human observers and the parameters of the constants of these functions [26]. To compare our results to predictions from *prospect theory* in the literature, we re-plotted our data with the methods in these reports, which use a logit function to fit curves to the difference in perceived value V(x,p) (see Appendix S1). Logit functions give similar results to linear regression of normalized data [25]. To avoid circularity in the results, we used parameters for these non-linear functions estimated from an independent sample of healthy observers in another study, which were also shown to be comparable to the results from a substantial number of other reports from healthy subjects [26].

**Figure 3:**
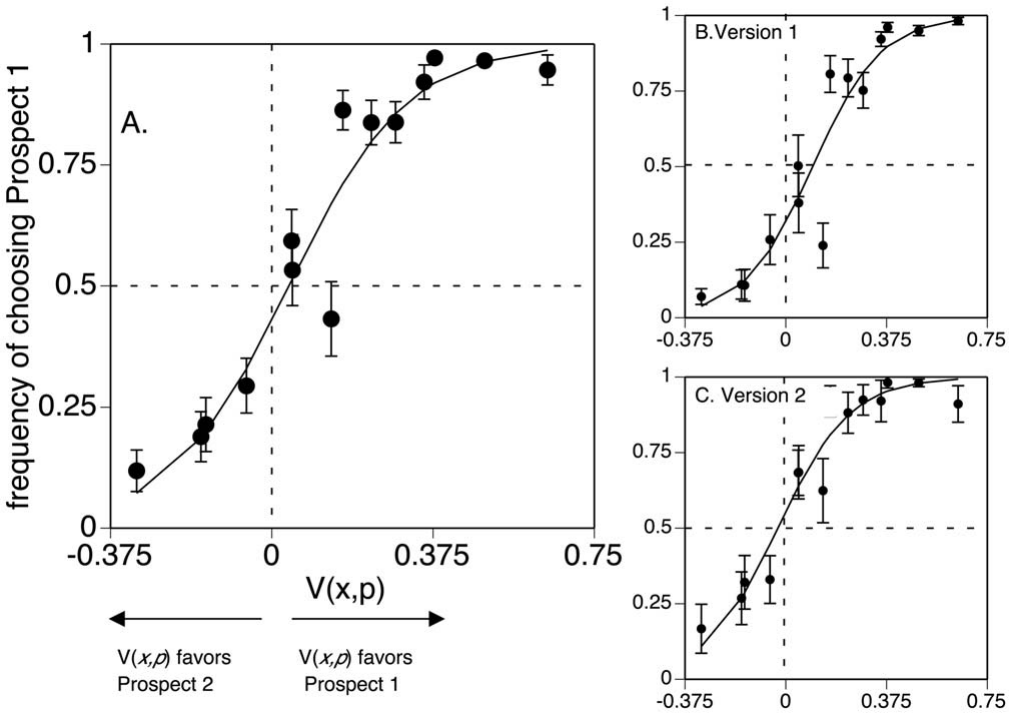
Results plotted in *prospect theory*terms. The frequency of choosing Prospect 1 is plotted as function of the difference in *perceived value* (V(x,p), see Appendix S1) rather than objective value, for all subjects (graph A), subjects in Version 1 (graph B), and subjects in Version 2 (graph C). Values greater than zero indicate that Prospect 1, with the higher reward probability, has the greater perceived value. Compared to Figure 2, the fitted logit function for all subjects now passes through the line of equivalence (frequency = 0.5) close to where V(x,p) = 0. This is because V(x,p) = 0 at a negative value of EV-ratio, due to the non-linearites in perceived utility and perceived probability (see Appendix S1, Image I-right graph). Error bars are one standard error.

One of the byproducts of these non-linear functions is that the predicted point of equivalence for perceived value, a subjective judgment, does not coincide with the point of equivalence for (objective) expected value, but rather occurs to its left. Figure 3 shows that when we re-plotted our data in terms of the difference in perceived value, using logit functions, these curves now pass close to zero. Hence, *prospect theory* can explain why subjects tend to favour prospects with higher probability over prospects with larger rewards, as seen in Figure 2.

## Discussion

Our paradigm examined decision-making under risk by requiring subjects to select between one prospect with higher probability of gain and another with higher magnitude of gain. First, we found that a change of 43% in the expected value ratio was required to shift responses from the point of equanimity, where subjects were equally likely to choose either option, to a 75% likelihood of selecting the prospect with higher expected value: this can be considered a *“value-threshold”* for decision-making. Second, our results showed a choice bias: the point of equanimity occurred not when both prospects had equal expected value, but when the prospect with the higher magnitude of gain had 38% more expected value than the prospect with higher probability. In other words, there was a tendency for subjects to choose the ‘safer’ (i.e. higher probability) prospect, which was outweighed only when the less likely proposition had an expected value of 38% or more than that of the safer prospect.

A number of studies have used a similar paradigm that requires subjects to choose between prospects varying in probability and magnitude of reward. This has been done to compare the validity of different decision-making models [27], to derive parameters for fitting and evaluating different functions in *prospect theory* (see [26] for review), to compare the predictions of prospect theory using different levels of incentives [28], or to examine effects that occur at the stage of input and selection [26], [29]. Others have used this type of experimental design to investigate the correlates of the different choice variables with neuroimaging. For example, functional MRI studies have suggested that there are distinct neural representations for the value of a prospect and for its uncertainty [19], [20], as well as an aggregate signal that encodes both [30]. Different regions are activated depending on whether a subject is making a decision under ambiguity or under risk [18], though the value of a reward is represented by a common signal in both cases [13]. Other studies have found a neural correlate for subjective risk - that is, the degree of risk-aversion [12]. Distinct neural substrates for different decision-making factors has also been shown with sequential choice versions [31], and in understanding the ‘exploration-exploitation’ dilemma relevant to learning [32], [33].

Thus, our simple experimental design has an extensive history and acceptance as a valid and valuable method of exploring decisions under risk. Our results show that this type of paradigm can also be used to behaviourally characterize and quantify sensitivity and bias in choice in a sample of human subjects. Sensitivity and bias are common summary variables in studies of perceptual processing: here we show that they may also be useful measures of a cognitive evaluation of risk, and potentially applicable to the study of decision-making in clinical populations. In our group of unselected healthy young subjects, our parameters were fairly robust and consistent across subjects, with 95% confidence intervals of [0.27, 0.59] for discriminative threshold and [−0.71, −0.05]. for decision bias. Hence it should be feasible to employ this design to study disordered patient groups.

Our chief goal was to characterize human decisions under risk as a function of the objective values of choices presented. As such, this employs the framework of *expected value theory*, which holds that the expected value of a choice is the product of the magnitude and probability of reward for that choice. A thoroughly rational ‘ideal evaluator’ in this situation would simply select the prospect with the largest expected value: in place of the sigmoid-shaped curve in Figures 2 and 3, there would be a step-function at an EV-ratio of zero: the infinite slope of a step function would correspond to a discriminative threshold of zero (i.e. an ‘ideal evaluator’ would be exquisitely sensitive to any change in EV), and its occurrence at an EV-ratio of zero would indicate no bias toward magnitude or probability in the decision, in other words, a neutral risk attitude that is neither avoiding nor seeking risk. Our finding that the discriminative threshold is 43% - i.e. that there is a sigmoid-shaped curve - reflects limits in our subjects' ability to estimate small differences in expected value, which in some models is represented as deriving from a stochastic choice variable [24]. The existence of a choice bias showing a risk-premium of 38% of expected value indicates that human behaviour deviates from an ideal evaluation of the objective value of prospects and is not risk-neutral but rather, risk-averse.

*Expected value theory* remains a widely used decision-making model, in part because of its simplicity. Experiments performed in primates largely base their analysis of reward processing on its predictions [4], [8], [34], it is used to model probabilistic decision-making in human behavioral studies [7], [9], [35], [36] and human imaging work [20], [21] and it forms the implied basis of the widely used Iowa gambling task [14]. However, the fact that humans deviate from rationality in decision-making under risk has been known for some time, and was the impetus for the development of models superseding expected-value theory. Two limitations of expected-value theory have been highlighted in particular. First, it does not take into account the utility of the outcome or the effect of decreasing marginal sensitivity. That is, it assumes that all subjects value outcomes (gains or losses) uniformly, irrespective of factors like their pre-existing wealth or the gains already accumulated: for example, a gain of $1000 is probably more meaningful to someone with nothing than to someone who has already won $1,000,000. *Expected utility theory* corrected this with a non-linear utility function for outcomes [26], [37]. The second limitation of *expected value theory* is that individual risk-aversion or risk-seeking tendencies may affect how subjects weigh probability information in their decision: *mean variance theory* handles this by weighting the payoff by their susceptibility to risk [38]. *Prospect theory* is the most recent model and it uses a different, more integrated approach to risk behaviour to address these limitations [22], [39]. The basic tenet of *prospect theory* is that subjects evaluate choice not on the basis of objective estimates of expected value, but by their subjective perception of value, which has inherent systematic biases. The perception of reward magnitude is characterized by an exponential function in which increments in reward are valued more at low than at high levels of reward (thus the difference between four and five dollars is less meaningful than the difference between one and two dollars). The perception of probability is characterized by a non-linear S-shaped weighting function that adjusts for our tendency to overweight low probabilities and underweight high probabilities. Such adjustments were motivated by anomalous human behaviour at these extremes, as manifest in decisions to buy insurance against low-probability losses or to buy lottery tickets for low-probability wins, for example [40]. Individual risk behaviour thus depends on both the probability weighting function and the non-linearity of the utility function [41] such that, according to *prospect theory*, people tend to be risk-averse for high probability gains and risk-seeking for low probability gains [39]. This differs from the mean-variance approach to decision-making, which takes into account individual risk susceptibility by adjusting risk (the variance of outcomes) for an individual's subjective sense of risk [42].

In our study, the anomalous behavior is the choice bias of 38%, indicating a tendency to favor a prospect with slightly higher probability over one with slightly higher reward. This bias can be viewed as another way of expressing “risk premium”. Risk premium is considered the amount of expected value that subjects are willing to forego to avoid risk, and is often operationally defined as the difference in expected value between the gain a subject is willing to take with 100% certainty and a gamble with uncertainty [43]. Therefore, if a subject is willing to take $45 with 100% certainty as opposed to a bet for $100 with 50% probability, the risk premium is $5 = $50(0.5)−$45(1.0). This has also been termed the ‘certainty equivalent’ [22] and has been calculated to compare risk behaviours across groups [44]. One possible limitation of this measure is that it is often derived from hypothetical choices [12] that do not necessarily reflect real choices with consequences (e.g.. monetary pay-outs) [28]. In our study, we quantified risk premium as a proportion of the expected value of the prospect under consideration: as such it is a relative value and can transfer to other choice currencies. In addition, it derived from real choices the subjects faced, which confers an important element of affective engagement, as well as real-life validity. Risk premium is not explained by *expected value theory* since it defies its basic axiom of rationality. However, using independently obtained estimates of parameters from other studies of healthy subjects, we could show that this risk premium is predicted as the product, not of objective measures of probability and size of gain, but of subjective measures of perceived probability and perceived utility of gain. Hence *prospect theory* may account not only for irrational human choices at the extremes of the probability spectrum, but also for anomalous choices with more typically encountered levels of probability.

An important feature of our experimental design is that the probabilities and magnitudes of reward associated with each choice were explicitly provided in each trial. As pointed out, this created a situation of risk, but importantly, this also eliminated the need for subjects to learn these parameters. This differs from several studies assessing behavior as a function of expected value [7], [21] and current clinical paradigms such as the Iowa gambling task [14], [45] and the Balloon Analogue Risk-Taking task [17]. For example, in the Iowa Gambling Task, subjects choose cards from one of four decks. There is a gain with every card, which is identical for all cards in a specific deck; however, the two decks with the higher gains also have the risk of occasional high losses, so that in the long run it is more advantageous to select from the decks with modest gains. Healthy participants quickly discover that the decks with lower gains are most advantageous, but certain disease populations fail to do this [46], [47], [48]. As subjects do not start with information about the gains and losses of each deck, they must learn the ‘tortoise-and-hare’ moralistic dimension of this paradigm over the course of the test. As a result, whether poor performance on the Iowa gambling task reflects a deficit in reward-related decision-making or impaired learning remains contentious [15]. This could be an important confound as patient populations suspected of having impaired decision-making under risk may have cognitive dysfunction that also impacts their learning and/or their reaction to uncertainty.

Given the prominence of the Iowa Gambling Test, it is worth highlighting other important differences between this test and our paradigm. There are only two expected values operating in the Iowa Gambling Test, a higher one for the two decks with lower gains and a lower one for the two with higher gains. Thus it cannot quantify the sensitivity of subjects to differences in expected value. Second, although the decks differ in the probability of losses, the decks with higher probability of loss have the same expected value as those with lower probability. Hence the test cannot assess how subjects incorporate probability information in their decision-making. In contrast, our paradigm was designed to study how subjects balance explicit information about the probability of gain against the magnitude of gain, and by doing so over a range of values, provides an estimation of the sensitivity of the subject to expected value.

It has been stated that new tests that specifically examine decisions under risk are needed to complement the assessment of how subjects learn loss-aversive strategies in the Iowa Gambling Task [16]. Also, though widely accepted, *prospect theory* has seldom been used as a framework for evaluating decisions in clinical populations, even in those with established anomalies in reward processing. For example, patients with Parkinson's disease have been shown to be less sensitive to reward (positive feedback) as a result of their dopamine-depleted state [49], [50], [51] but become less sensitive to penalty (negative feedback) when treated with dopamine agonists [47], [52], [53]. Dopamine agonist treatment can also turn some patients with Parkinson's disease into pathologic gamblers [54]. Studies using functional MRI have demonstrated that patients with an impulse control disorder while on dopamine agonists show both a behavioural bias towards risky choices and reduced neural activation by risk, compared to controls with Parkinson's disease but no impulse control problems [55]. At this point, however, it is not known whether these patients are compelled to bet by an altered perception of risk as would be suggested by the mean-variance approach to risk behaviour [55] or if their deficit can be attributed to one of the decision-making variables described by *prospect theory*: either an inflated perception of gains or an over-estimation of small probabilities of winning. As seen in the Appendix S1, *prospect theory* makes very different predictions about the effects on discriminative thresholds from these two different manipulations.

In conclusion, we showed that this simple test provides a robust measure of discriminative value thresholds and biases in decision-making under risk, in a design that eliminates confounds of decisional ambiguity and learning. We show that healthy subjects show a choice bias that favours probability over magnitude, which can be explained by non-linearities in the subjective perception of the value of a choice, as predicted by *prospect theory*. *Prospect theory* makes predictions about the effect of changing parameters in these non-linear functions, which may generate useful insights when used to evaluate the decisions under risk of populations with anomalous reward-processing, such as Parkinson's disease and schizophrenia.

## Supporting Information

Appendix S1 Explanation of *Prospect theory* and its predictions.
(PDF)

Appendix S2 Application of our paradigm using manual responses.
(PDF)

## References

[1] Platt M, Huettel S (2008). Risky business: the neuroeconomics of decision making under uncertainty.. Nat Neurosci.

[2] Schultz W (2002). Getting formal with dopamine and reward.. Neuron.

[3] Canavan AG, Passingham RE, Marsden CD, Quinn N, Wyke M (1989). The performance on learning tasks of patients in the early stages of Parkinson's disease.. Neuropsychologia.

[4] Hikosaka O, Nakamura K, Nakahara H (2006). Basal ganglia orient eyes to reward.. J Neurophysiol.

[5] Dunn BD, Dalgleish T, Lawrence AD (2006). The somatic marker hypothesis: a critical evaluation.. Neurosci Biobehav Rev.

[6] Yechiam E, Busemeyer JR, Stout JC, Bechara A (2005). Using cognitive models to map relations between neuropsychological disorders and human decision-making deficits.. Psychol Sci.

[7] Milstein DM, Dorris MC (2007). The Influence of Expected Value on Saccadic Preparation.. J Neurosci.

[8] Ding L, Hikosaka O (2007). Temporal development of asymmetric reward-induced bias in macaques.. J Neurophysiol.

[9] Blaukopf CL, DiGirolamo GJ (2006). Differential effects of reward and punishment on conscious and unconscious eye movements.. Exp Brain Res.

[10] Roesch MR, Olson CR (2004). Neuronal activity related to reward value and motivation in primate frontal cortex.. Science.

[11] Ross M, Lanyon LJ, Viswanathan J, Manoach DS, Barton JJS (2011). Human prosaccades and antisaccades under risk: effects of penalties and rewards on visual selection and action value.. Neuroscience.

[12] Christopoulos GI, Tobler PN, Bossaerts P, Dolan RJ, Schultz W (2009). Neural correlates of value, risk, and risk aversion contributing to decision making under risk.. J Neurosci.

[13] Levy I, Snell J, Nelson AJ, Rustichini A, Glimcher PW (2010). Neural representation of subjective value under risk and ambiguity.. J Neurophysiol.

[14] Bechara A, Damasio AR, Damasio H, Anderson SW (1994). Insensitivity to future consequences following damage to human prefrontal cortex.. Cognition.

[15] Toplak ME, Sorge GB, Benoit A, West RF, Stanovich KE (2010). Decision-making and cognitive abilities: A review of associations between Iowa Gambling Task performance, executive functions, and intelligence.. Clin Psychol Rev.

[16] Schonberg T, Fox CR, Poldrack RA (2011). Mind the gap: bridging economic and naturalistic risk-taking with cognitive neuroscience.. Trends Cogn Sci.

[17] Lejuez CW, Read JP, Kahler CW, Richards JB, Ramsey SE (2002). Evaluation of a behavioral measure of risk taking: the Balloon Analogue Risk Task (BART).. J Exp Psychol Appl.

[18] Huettel SA, Stowe CJ, Gordon EM, Warner BT, Platt ML (2006). Neural signatures of economic preferences for risk and ambiguity.. Neuron.

[19] Schultz W, Preuschoff K, Camerer C, Hsu M, Fiorillo CD (2008). Explicit neural signals reflecting reward uncertainty.. Philos Trans R Soc Lond B Biol Sci.

[20] Tobler PN, O'Doherty JP, Dolan RJ, Schultz W (2007). Reward value coding distinct from risk attitude-related uncertainty coding in human reward systems.. J Neurophysiol.

[21] Rolls ET, McCabe C, Redoute J (2008). Expected value, reward outcome, and temporal difference error representations in a probabilistic decision task.. Cereb Cortex.

[22] Kahneman D, Tversky A (1979). Prospect theory: an analysis of decision under risk.. Econometrica.

[23] Lesieur H, Blume S (1987). The South Oaks Gambling Screen (SOGS): a new instrument for the identification of pathological gamblers.. A– J Psychiatry.

[24] Stott H (2006). Cumulative prospect theory's functional menagerie.. Journal of Risk and uncertainty.

[25] Simpson T (1995). Vision Thresholds from psychometric analyses: alternatives to probit analysis.. Optometry Vision Sci.

[26] Hsu M, Krajbich I, Zhao C, Camerer CF (2009). Neural response to reward anticipation under risk is nonlinear in probabilities.. J Neurosci.

[27] Camerer C (1989). An experimental test of several generalized utility theories.. Journal of Risk and Uncertainty.

[28] Holt C, Laury S (2002). Risk aversion and incentive effects.. American Economic Review.

[29] Tobler PN, Christopoulos GI, O'Doherty JP, Dolan RJ, Schultz W (2008). Neuronal distortions of reward probability without choice.. J Neurosci.

[30] Tobler PN, Christopoulos GI, O'Doherty JP, Dolan RJ, Schultz W (2009). Risk-dependent reward value signal in human prefrontal cortex.. Proc Natl Acad Sci USA.

[31] Symmonds M, Bossaerts P, Dolan RJ (2010). A behavioral and neural evaluation of prospective decision-making under risk.. J Neurosci.

[32] Daw ND, O'Doherty JP, Dayan P, Seymour B, Dolan RJ (2006). Cortical substrates for exploratory decisions in humans.. Nature.

[33] Payzan-LeNestour E, Bossaerts P (2011). Risk, unexpected uncertainty, and estimation uncertainty: Bayesian learning in unstable settings.. PLoS Comput Biol.

[34] O'Neill M, Schultz W (2010). Coding of reward risk by orbitofrontal neurons is mostly distinct from coding of reward value.. Neuron.

[35] Liston D, Stone L (2008). Effects of prior information and reward on oculomotor and perceptual choices.. J Neurosci.

[36] Stritzke M, Trommershäuser J, Gegenfurtner K (2009). Effects of salience and reward information during saccadic decisions under risk.. Journal of the Optical Society of America A.

[37] Savage L (1954). The foundation of statistics.

[38] Markowitz H (1952). Portfolio Selection.. Journal of Finance.

[39] Fox C, Poldrack R (2009). Prospect theory and the brain.. Neuroeconomics Decision Making and the Brain.

[40] Camerer C (2000). Neuroeconomics Decision Making and the BrainProspect theory in the wild: Evidence from the field; Kahneman D, Tversky A, editors.

[41] Glimcher PW (2008). Understanding risk: A guide for the perplexed.. Cogn Affect Behav Neurosci.

[42] D'Acremont M, Bossaerts P (2008). Neurobiological studies of risk assessment: a comparison of expected utility and mean-variance approaches.. Cogn Affect Behav Neurosci.

[43] Weber E, Johnson E (2008). Decisions under uncertainty: Psychological, economic, and neuroeconomic explanations of risk preference.

[44] Stanton SJ, Mullette-Gillman OA, McLaurin RE, Kuhn CM, LaBar KS (2011). Low- and high-testosterone individuals exhibit decreased aversion to economic risk.. Psychol Sci.

[45] Bechara A, Tranel D, Damasio H, Damasio AR (1996). Failure to respond autonomically to anticipated future outcomes following damage to prefrontal cortex.. Cereb Cortex.

[46] Shurman B, Horan WP, Nuechterlein KH (2005). Schizophrenia patients demonstrate a distinctive pattern of decision-making impairment on the Iowa Gambling Task.. Schizophr Res.

[47] Pagonabarraga J, Garcia-Sanchez C, Llebaria G, Pascual-Sedano B, Gironell A (2007). Controlled study of decision-making and cognitive impairment in Parkinson's disease.. Mov Disord.

[48] Kobayakawa M, Koyama S, Mimura M, Kawamura M (2008). Decision making in Parkinson's disease: Analysis of behavioral and physiological patterns in the Iowa gambling task.. Mov Disord.

[49] Menza MA, Golbe LI, Cody RA, Forman NE (1993). Dopamine-related personality traits in Parkinson's disease.. Neurology.

[50] Ragonese P, Salemi G, Morgante L, Aridon P, Epifanio A (2003). A case-control study on cigarette, alcohol, and coffee consumption preceding Parkinson's disease.. Neuroepidemiology.

[51] Tomer R, Aharon-Peretz J (2004). Novelty seeking and harm avoidance in Parkinson's disease: effects of asymmetric dopamine deficiency.. J Neurol Neurosurg Psychiatry.

[52] Bodi N, Keri S, Nagy H, Moustafa A, Myers CE (2009). Reward-learning and the novelty-seeking personality: a between- and within-subjects study of the effects of dopamine agonists on young Parkinson's patients.. Brain.

[53] van Eimeren T, Ballanger B, Pellecchia G, Miyasaki JM, Lang AE (2009). Dopamine agonists diminish value sensitivity of the orbitofrontal cortex: a trigger for pathological gambling in Parkinson's disease?. Neuropsychopharmacology.

[54] Dodd ML, Klos KJ, Bower JH, Geda YE, Josephs KA (2005). Pathological gambling caused by drugs used to treat Parkinson disease.. Arch Neurol.

[55] Voon V, Gao J, Brezing C, Symmonds M, Ekanayake V (2011). Dopamine agonists and risk: impulse control disorders in Parkinson's disease.. Brain.

