# Supplementary material for: Sensitivity and Bias in Decision-Making under Risk: Evaluating the Perception of Reward, Its Probability and Value
Source: PLoS One. 2012 Apr 6;7(4):e33460. doi: 10.1371/journal.pone.0033460 (PMC3320893; doi:10.1371/journal.pone.0033460)
Supplement: Appendix S1 — Explanation of Prospect theory and its predictions. (PDF) [file pone.0033460.s001.pdf]

APPENDIX S1

In *prospect theory*, choices are made not by objective measures of probability and magnitude as in *expected value theory*, but by subjective perception of these variables, which show important non-linearities that are captured by weighting functions [22].

For the reward at stake, subjects consider the utility of the gain v(x), rather than its absolute magnitude x. The most common weighting function used to model utility is a power function (Image I, left):

$$v(x) = x^{\rho} \qquad (1)$$

As the sizes of rewards increase, further increments in reward are generally considered less valuable, and so ρ is usually <1.

For the probability of reward, a single-parameter Prelec function [26] has been shown to capture adequately the tendency of humans to overestimate small probabilities and underestimate large ones (Image I, middle):

$$w(p) = \exp(-(-\ln p)^{\alpha} \qquad (2)$$

These perceived probabilities and utilities are combined multiplicatively as in standard utility theory, to give the function for U(L), the perceived value of prospect L:

$$U(L) = v(x)\, w(p) \qquad (3)$$

The difference between the perceived value of two prospects L_1 and L_2 is then simply:

$$V(x,p) = U(L_1)-U(L_2) \qquad (4)$$

When choosing between these two prospects, the likelihood that a subject will choose the first prospect (P(L_1) can be represented by a logit function:

$$P(L_1)=1/(1+\exp\{-\lambda(V(x,p))\}) \qquad (5)$$

The parameter λ is introduced as a stochastic constant that controls the steepness of the function [24], which in turn reflects the subject's sensitivity to the difference in perceived value between L_1 and L_2.

To model our experimental parameters in *prospect theory*, we used constants for Equations 1 and 2 derived from independent studies of other healthy populations, as reviewed comprehensively elsewhere [26]. For the utility function (1), we used $\rho = 0.57$; for the Prelec function, we used $\alpha = 0.77$. With these constants, the relation between the

V(*x,p*) function of *prospect theory* and the EV-ratio of *expected value theory* is depicted in Image I (right). While there is an approximately linear relation between the two, a key finding is that the point of equivalence between perceived value of the two prospects (when V(*x,p*) = 0) occurs when the EV-ratio is negative. Hence *prospect theory* with current best estimates of the parameters in its non-linear functions predicts a choice bias in favour of prospects with higher probability over prospects with larger reward.

This suggests that the choice bias we found when our parameters are expressed as an EV-ratio may be minimized or eliminated if they are expressed as V(*x,p*). However, because $P(L_1)$ is constrained in equation 4 to have an equivalence point of zero, since $P(L_1) = 0.5$ when $U(L_1) = U(L_2)$, we added a second 'intercept constant' k to the equation:

$$P(L_1)=1/(1+\exp\{-\lambda(V(x,p)+k)\}) \qquad (6)$$

If the best fit to our data is found when k = 0, this would suggest that the choice bias we found can be attributed to the non-linearities of equations 2 and 3. Hence we estimated the stochastic constant λ and the intercept constant k by least-squares fit of curve predictions to the actual data. The best fit to the group data was obtained with $\lambda = 7.2$ and $k = 0.04$ (see Figure 3 in main text): the choice bias seen when plotted against EV-ratio is nearly eliminated when the data are re-plotted in terms of differences in perceived value V(*x,p*).

We can also consider hypothetical impact of disease states on the parameters of *prospect theory*. In pathological gambling, one might surmise that subjects are more prone to choose a prospect with larger reward despite its low probability. This could occur because of distorted perceptions of either gain or probability. We can model the first by showing a family of utility functions (1) v(*x*), with ρ increasing from 0.8 to 1.4, while the α parameter in the perceived probability function (2) w(*p*) is held constant at 0.77 (Image II). When we plot the predicted choices made at different EV-ratios, we find that the curves gradually shift to the right as ρ increases, indicating greater likelihood of choosing the prospect with larger reward.

We can also model the converse, the anticipated effects of altered perceptions of probability. We can model a family of Prelec functions (2) w(*p*) for perceived probability, with α declining from 0.7 to 0.25, while ρ in the utility function (1) v(*x*) is held constant

at 0.57. Note that, while there is an increasingly inflated perception for low probabilities as α decreases, there is also a progressive flattening of the slope of the Prelec function in the mid-range of probabilities. Again, we find that for predicted choice as a function of EV-ratios, the curves shift to the right, indicating a shift to choosing the prospect with larger gain.

Thus, a tendency to choose the side with greater reward can occur as the result of either increasing the exponential term in the utility function (1) v(x), or decreasing the exponential term in the Prelec function for perceived probability (2) w(*p*). However, the difference between the two is that the change in the utility function for reward size also leads to steeper slopes, and therefore increased sensitivity to differences in EV-ratio, whereas the change in the probability function leads to shallower slopes, and therefore reduced sensitivity, likely because of the flattening of the Prelec function in the mid-range. Hence these two scenarios in *prospect theory* predict diametrically opposite effects on discriminative thresholds, which leads to testable predictions in patient studies.

Finally it is also possible to model individual subject performance with prospect theory [26,44]. We used a three-parameter-fit iterative procedure to find optimal estimates of ρ, α, and λ for each subject's data, with the aim of minimizing the summed variance in both slope and intercept between the logit function and the psychometric function fitted to the data, in z-transformed space. This showed that for the utility function of $v(x) = x^{\rho}$ the mean value was 0.58 (s.e. 0.03), for the Prelec function $w(p) = \exp(-(-\ln p)^{\alpha}$ the mean value of α was 0.63 (s.e. 0.03), and the mean value of the stochastic constant λ was 7.35 (s.e. 0.31). Others have compared points of equivalence with these best-fit values for the exponential functions and found a correlation [44]. As seen in Image II, one would expect a positive correlation between ρ and the point of equivalence, as the latter shifts rightward as ρ increases. In our subjects (Image III), we did find a significant positive correlation of $r = .73$ ($F(1,17) = 19.2$, $p<.0005$). Image II would also predict a negative correlation between α, and the point of equivalence: we did find a negative slope for this relationship, although the correlation was not significant ($r = -0.37$, $F(1,17) = 2.77$, $p = 0.11$). These data

would suggest that the major determinant of the equivalent point is the utility function.

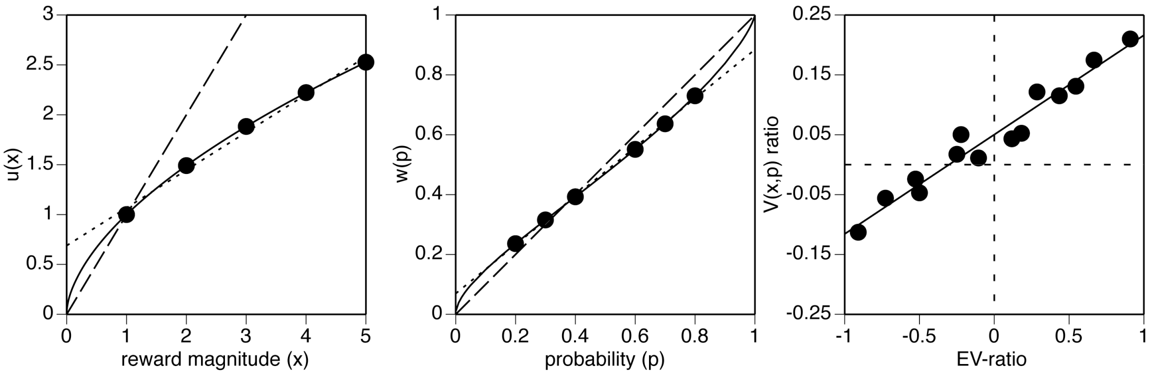

Image I. *Perceived* magnitude, probability and value using the non-linear weighting functions of *prospect theory*. (A) Perceived utility of reward v(x) is plotted against reward magnitude x, showing decreasing marginal sensitivity with the perception of magnitude decreasing as value increases. (B) Perceived probability w(p) plotted against reward probability p using the single-parameter Prelec function demonstrates over-estimation of low probabilities and underestimation of high probabilities. In (A) and (B), solid lines indicate the non-linear functions of *prospect theory*, while the dashed lines indicate the simple linear predictions of *expected value theory* (i.e. v(x) = x, w(p) = p); black discs are the values used in our experiment. (C) Difference in perceived value V(x,p) plotted against EV-ratio shows that the points of equivalence for *perceived* value and EV (when these are zero, shown as the dotted lines) do not intercept: rather the point of equivalence for V(x,p) occurs when the EV-ratio is a negative value. Thus *prospect theory* predicts that humans favour higher probability over larger reward, even when the rational choice based on EV favours the latter: i.e. they are risk-averse.

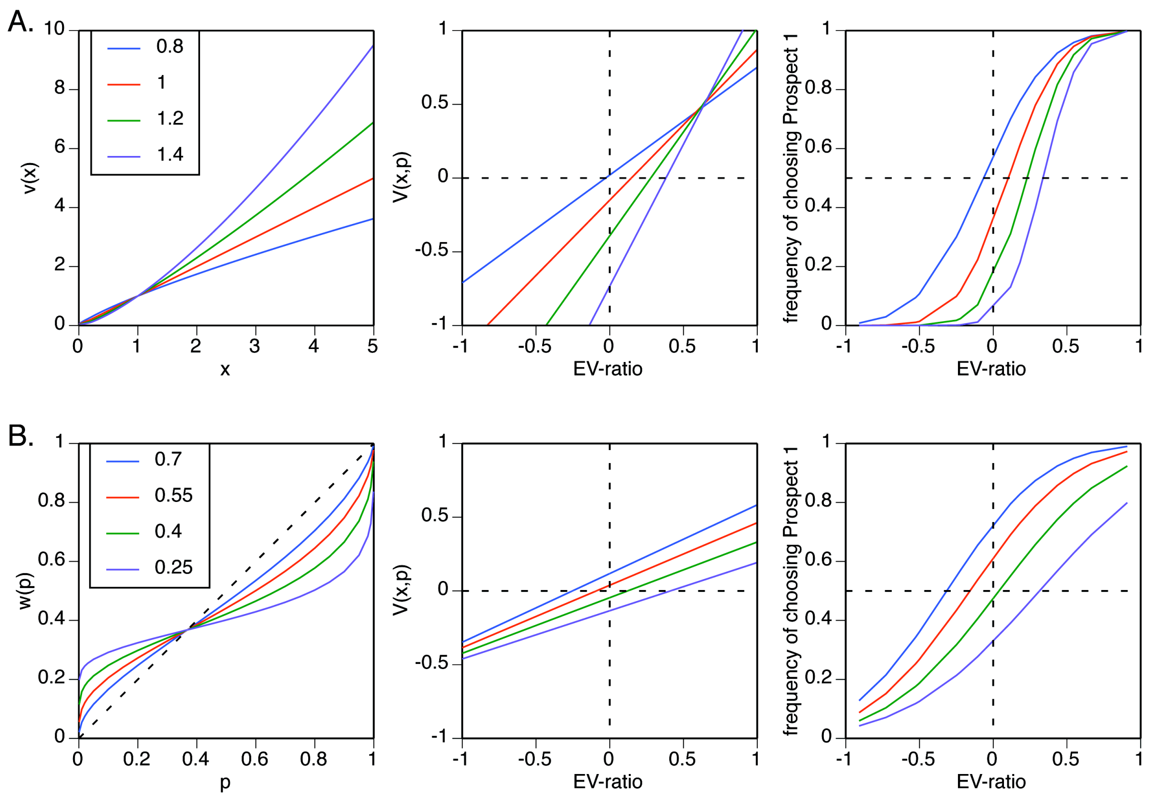

Image II. Hypothetical impact of disease states on parameters of choice in *prospect theory*. - how does one become a pathological gambler? (A) In the top row, heightened perceptions of magnitude of reward might lead to a dampening of the 'decreasing marginal sensitivity' effect and a steepening of the relationship between *perceived* magnitude v(x) and magnitude x (top, left) which would alter the relationship between *perceived* value difference V(x,p) and EV-ratio (top, middle) and shift the curve of predicted choice to the right, indicating a tendency to choose prospects with the higher reward magnitude (top, right). (B) In the bottom row, increasing the non-linear relationship between *perceived* probability w(p) and objective probability p distorts the extremes of probability so subjects over-estimate low probabilities but at the price of decreased sensitivity to changes in the mid-range (bottom, left), altering the relationship between *perceived* value difference V(x,p) and EV-ratio (bottom, middle) and also shift the curve of predicted choice to the right, again indicating a tendency to choose prospects with larger gain (bottom, right). However, in (A) the slopes steepen from left to right (top right), with heightened thresholds to expected value, while in (B) the slopes flatten (bottom right), corresponding to degraded thresholds for expected value.

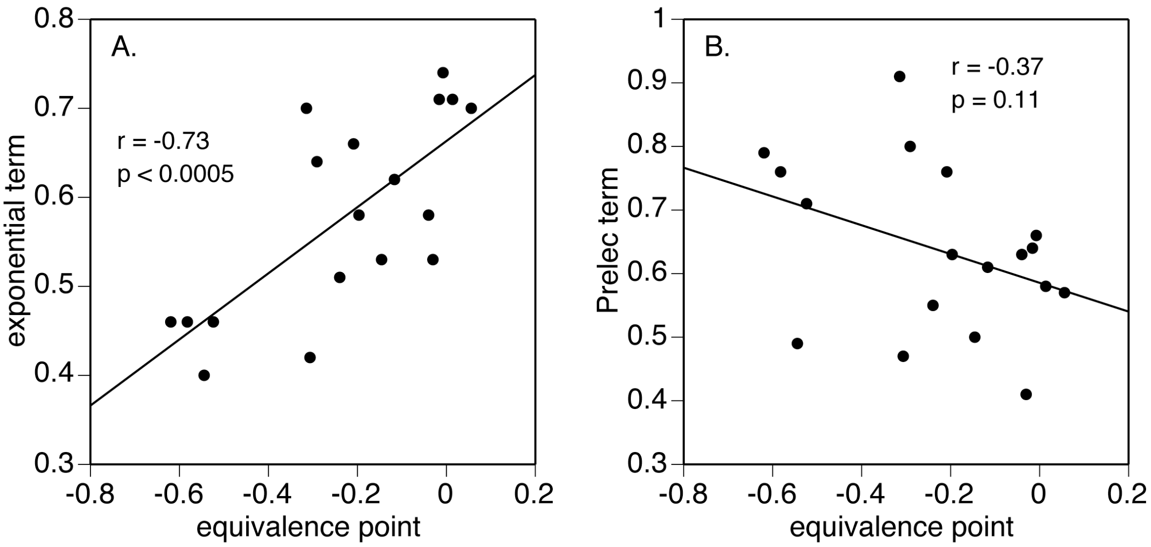

Image III. Relationship between parameters in prospect theory and points of equivalence for individuals. A. Individual estimates of ρ (‘exponential term’) in the utility function v(x) = x^{ρ} for perception of reward magnitude, as a function of equivalence point for each subject. B. Individual estimates of α (‘Prelec term’) in the Prelec function w(p) = exp(-(-ln$p$)$^{\alpha}$ for perception of reward probability, as a function of equivalence point for each subject. Correlation values (r) and their significance (p) are indicated.
