# Supplementary material for: Sensitivity and Bias in Decision-Making under Risk: Evaluating the Perception of Reward, Its Probability and Value
Source: PLoS One. 2012 Apr 6;7(4):e33460. doi: 10.1371/journal.pone.0033460 (PMC3320893; doi:10.1371/journal.pone.0033460)
Supplement: Appendix S2 — Application of our paradigm using manual responses. (PDF) [file pone.0033460.s002.pdf]

APPENDIX S2

One reviewer asked if this paradigm would also work with manual keypresses. We show (Image IV) that application of this same paradigm to a group of healthy elderly controls in another experiment yields data with a similar configuration, which can also be fitted with the same type of psychometric function.

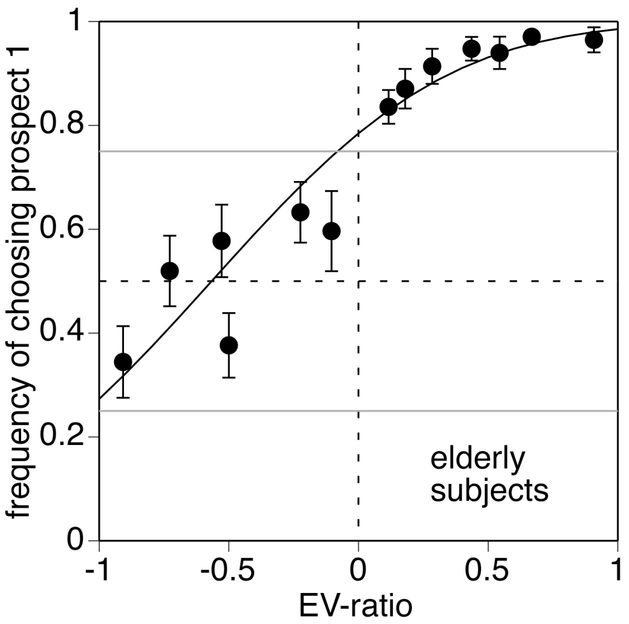

Image IV. Application of our paradigm using manual responses. The frequency of choosing Prospect 1 (the prospect with higher reward probability) is plotted as a function of the EV-ratio for 17 healthy subjects aged 55 to 75 years, with conventions similar to Figure 2A.
